# Persistent Head and Neck Cancer Following First-Line Treatment

**DOI:** 10.3390/cancers10110421

**Published:** 2018-11-03

**Authors:** Teresa Bernadette Steinbichler, Madeleine Lichtenecker, Maria Anegg, Daniel Dejaco, Barbara Kofler, Volker Hans Schartinger, Maria-Therese Kasseroler, Britta Forthuber, Andrea Posch, Herbert Riechelmann

**Affiliations:** 1 Department of Otorhinolaryngology-Head & Neck Surgery, Medical University of Innsbruck, Anichstr. 35, 6020 Innsbruck, Austria; madeleine.lichtenecker@student.i-med.ac.at (M.L.); maria.anegg@student.i-med.ac.at (M.A.); daniel.dejaco@i-med.ac.at (D.D.); ba.kofler@tirol-kliniken.at (B.K.); volker.schartinger@i-med.ac.at (V.H.S.); herbert.riechelmann@i-med.ac.at (H.R.); 2 Department of Internal Medicine V, Medical University of Innsbruck, Anichstr. 35, 6020 Innsbruck, Austria; marie-therese.kasseroler@tirol-kliniken.at; 3 Department of Therapeutic Radiology and Oncology, Medical University of Innsbruck, Anichstr. 35, 6020 Innsbruck, Austria; britta.forthuber@tirol-kliniken.at (B.F.); andrea.posch@tirol-kliniken.at (A.P.)

**Keywords:** persistent disease, salvage surgery, SBRT (stereotactic body radiotherapy), second-line treatment, neck dissection, complete response, best supportive care

## Abstract

*Background:* Following first-line treatment of head and neck cancer (HNC), persistent disease may require second-line treatment. *Methods:* All patients with HNC treated between 2008 and 2016 were included. Second-line treatment modalities and survival of patients were analyzed. *Results:* After first-line therapy, 175/741 patients had persistent disease. Of these, 112 were considered eligible for second-line treatment. Second-line treatment resulted in 50% complete response. Median overall survival of patients receiving second-line therapy was 24 (95% CI: 19 to 29) months; otherwise survival was 10 (9 to 11; *p* < 0.0001) months. Patients receiving second-line surgery had a median overall survival of 45 (28 to 62) months, patients receiving second-line radiotherapy had a median overall survival of 37 (0 to 79; *p* = 0.17) months, and patients receiving systemic therapy had a median overall survival of 13 (10 to 16; *p* < 0.001) months. Patients with persistent HNC in the neck had a better median survival (45 months; 16 to 74 months; *p* = 0.001) than patients with persistence at other sites. *Conclusion:* Early treatment response evaluation allows early initiation of second-line treatment and offers selected patients with persistent disease a realistic chance to achieve complete response after all. If possible, surgery or radiotherapy are preferable.

## 1. Introduction

The basic treatment aim in patients with head and neck cancer (HNC) is cure. This requires complete response (CR) to antitumor treatment [1]. This goal may not be achieved by first-line treatment and residual (RD) or progressive disease (PD) may persist. Patients with persistent disease may qualify for second-line treatment with the aim of reaching CR after all. Timely initiation of second-line treatment requires early detection of persistent disease by systematic evaluation of treatment response [2,3]. In our institution, response to first-line therapy is systematically evaluated 8–10 weeks following end of treatment. This interval is a compromise of continuing post-treatment tumor clearance and development of postradiogenic tissue fibrosis possibly interfering with surgical rescue. Moreover, this time interval allows radioresistant clones to proliferate and be detectable [4].

Treatment recommendations in HNC are provided by an interdisciplinary tumor board (ITB). In Austria, law regulates the organization of ITB in oncological centers. Head and neck ITBs require at least participation of a head and neck surgeon, a radiotherapist, an oncologist, a radiologist, and a pathologist. Common options of second-line treatment include surgery, radiotherapy (RT), systemic therapy including chemotherapy (CHT) and immunotherapy (IT), and combinations of these [5]. Patients who do not qualify for second-line treatment receive best supportive care (BSC) [6]. Several publications deal with specific second-line treatments, including salvage surgery [7] or innovative therapies [8], in highly selected patients with residual or recurrent disease. However, data on unselected patients with incident HNC failing first-line treatment under real-world conditions are rare. Moreover, there is limited information on which clinical parameters influence choice of second-line treatment by an ITB and on outcomes of different second-line treatment modalities.

This registry-based study intends to provide some information on these issues. Data of all patients with incident HNC treated between 2008 and 2016 were evaluated. Frequencies of RD or PD following first-line treatment and various factors influencing second-line treatment decisions by the ITB were assessed. Moreover, second-line treatment outcome for persistent HNC was investigated. Treatment of recurrent disease following a previous CR was not covered in this analysis.

## 2. Results

### 2.1. Tumor Registry Population, Exclusions, and Results of First-Line Treatment

**Figure 1:**
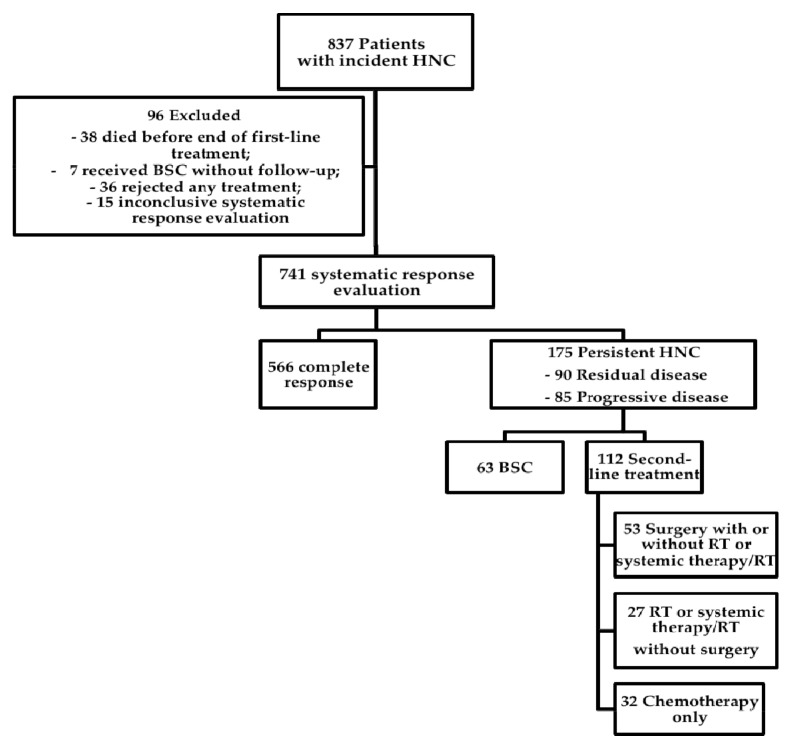
Overview of study population. Flow chart of the course of disease in 837 patients with incident head and neck cancer treated between 2008 and 2016 in a tertiary head and neck oncology center. A systematic response evaluation was performed 8–10 weeks following end of first-line treatment in 741 patients. Of these, 175 had persistent disease and were analyzed in detail. Residual disease included partial response and no change. Abbreviations: HNC: Head and neck cancer, BSC: Best supportive care, RT: Radiotherapy.

**Figure 2:**
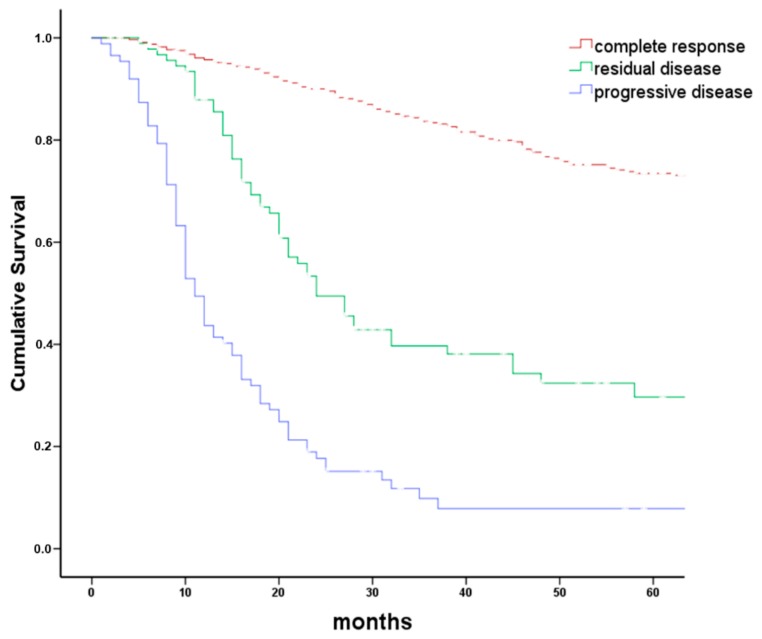
Overall survival grouped by tumor response to first-line therapy. Kaplan–Meier plot comparing overall survival in 741 patients with head and neck cancer grouped by response to first-line therapy. Treatment response was grouped in complete response (CR), residual disease (RD including partial response and no change), and tumor progression (PD). Logrank *p* < 0.001. Progressive disease was frequently associated with the new appearance of distant metastases.

From January 2008 to December 2016, 837 patients with incident HNC complying with inclusion criteria were recorded in the clinical tumor registry. Of these 837 patients, 96 were excluded (Figure 1). Reasons for exclusion were death before start or end of first-line treatment (*n* = 38), initial BSC without further follow-up (*n* = 7), refusal of any treatment (*n* = 36), or inconclusive systematic treatment response evaluation requiring further follow-up (*n* = 15). Of the 741 patients with conclusive systematic treatment response evaluation, 566 (76%) had CR and 175 (24%) had persistent disease following first-line treatment. Of these, 90 had RD (i.e., partial response or no change) and 85 had PD (Figure 1). Overall survival between patients with CR, RD, and PD differed significantly (Figure 2; log rank *p* < 0.001). Actuarial 5-year survival of patients with CR was 73 ± 3%, of patients with RD 21 ± 7%, and of patients with PD it was 3 ± 3%.

### 2.2. Characteristics of Patients with Persistent HNC

Of the 175 patients with persistent HNC following first-line treatment, 133 were male (Table 1). The mean age was 63 ± 1 years. Median follow-up was 54 (95% CI: 45–63) months. In patients with RD, the persistent tumor was frequently located at the primary tumor site and/or neck. Patients with PD following first-line treatment frequently had distant metastases (Table 2). In a Kaplan–Meier analysis, median survival of 32 patients with persistent disease only in the neck was better (45 months; 16–74 months; *p* = 0.001) than that of 143 patients with persistence at any other site. Median survival of patients with persistent disease at all other sites ranged between 12 and 20 months.

### 2.3. Best Supportive Care vs. Second-Line Treatment

**Figure 3:**
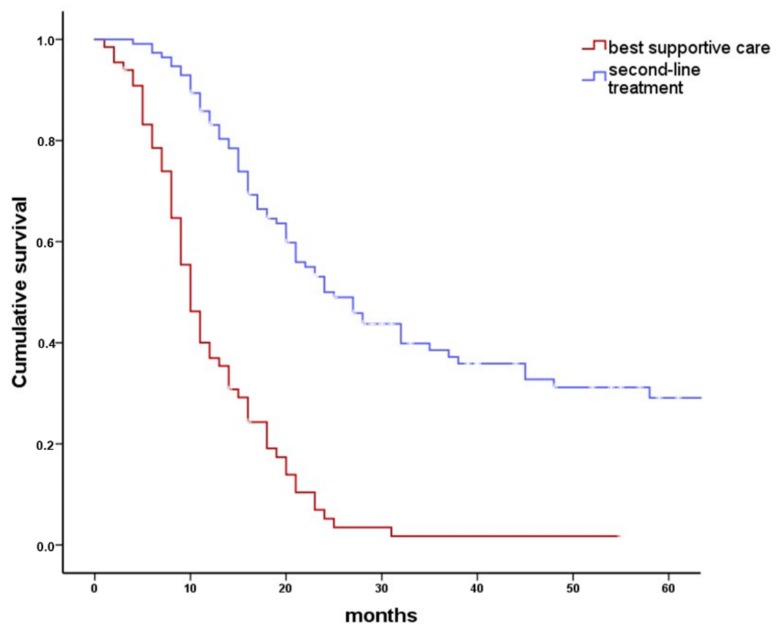
Overall survival after any second-line therapy compared to best supportive care (BSC). Kaplan–Meier plot comparing overall survival in 175 patients with persistent head and neck cancer who received either an antineoplastic second-line treatment or BSC. Selection of antineoplastic treatment was governed by the available treatment options remaining shortly after first-line treatment. Second-line treatment included surgical treatment, radiotherapy, systemic therapy, or combinations of these. Median overall survival was 10 months (95% CI: 9–11 months) for patients receiving BSC and 24 months (95% CI: 19–29 months) for patients receiving antineoplastic second-line therapy (log rank *p* < 0.001).

Of 175 patients with persistent HNC following first-line treatment, 63 (36%) were considered not to benefit from any second-line treatment by the ITB and received BSC (Figure 1). In a univariate chi-square frequency analysis (Table 3), factors influencing the ITB recommendation for or against second-line treatment included age at initial diagnosis (*p* < 0.001), American Society of Anesthesiology (ASA) score (*p* = 0.004), initial treatment adherence (*p* < 0.001), p16 status (*p* = 0.05), first-line treatment modality (*p* < 0.001), residual vs. progressive disease (*p* < 0.001), and site of persistence (*p* < 0.001). In Kaplan–Meier and actuarial analyses, median overall survival was significantly better in 112 patients receiving second-line therapy (24 months; 19–29 months) than in 63 patients receiving BSC (10 months; 9–11 months; *p* < 0.0001, Figure 3). Eighteen percent of the patients receiving second-line treatment survived more than five years (18 ± 6%). In contrast, no patient receiving BSC survived 5 years. The factors with impact on the ITB advice (Table 3) were included in a Cox regression model for overall survival. Back step elimination using a likelihood ratio of 0.05 for inclusion and 0.1 for exclusion was used. Patients receiving BSC had a three times higher risk of death than patients receiving any kind of second-line treatment (hazard ratio 3.2; 95% CI: 2.0–4.9; *p* < 0.001).

### 2.4. Second-Line Treatment Modalities

In 112 of 175 patients with persistent disease following first-line treatment, a second-line treatment was initiated. Due to their malignant disease, 12/112 patients (10%) died before the end of second-line therapy and 1 patient was lost to follow-up. Overall, 56/112 (50%) patients achieved CR following second-line therapy (Table 4). Persistence following second-line treatment was observed in 43/112 (38%) patients. These patients then received third-line systemic therapy or BSC.

Surgery was the most common second-line treatment modality (53/112, 47%). In 49 patients, surgery was the only second-line treatment and 4 patients received second-line surgery and postoperative RT or systemic therapy/RT. The most common type of second-line surgery was neck dissection (31/53, 57%), followed by surgery at the primary tumor site combined with neck dissection (12/53, 22%), second-line surgery at the primary tumor site alone (9/53, 17%) and other surgical treatment in 1 patient. CR was achieved in 41/53 (77%) patients treated with second-line surgery. Definitive RT or systemic therapy/RT without surgery was the second-line therapy in 27/112 patients (24%). Approximately half of these patients (14/27, 52%) achieved CR. Second-line systemic therapy was applied in 32/112 (29%) patients. Of these, 1 patient achieved CR.

**Figure 4:**
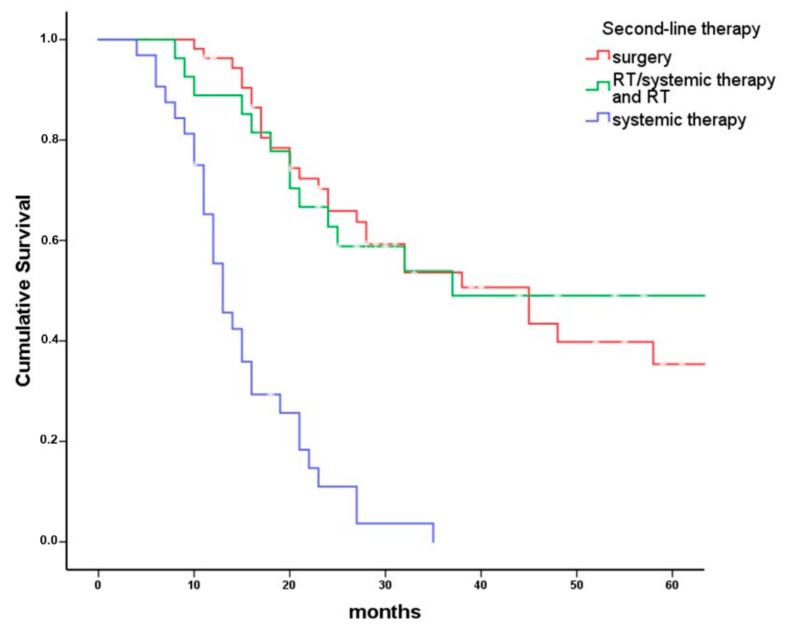
Overall survival of patients receiving second-line therapy for different treatment modalities. Kaplan–Meier plot comparing overall survival in 112 patients with persistent head and neck cancer grouped by second-line treatment modalities (surgery with or without RT or systemic therapy/RT *n* = 53, RT or systemic therapy/RT *n* = 27, systemic therapy only *n* = 32). Patients receiving surgery as second-line therapy had a median overall survival of 45 months (95% CI: 28–62 months). Patients receiving second-line RT with or without systemic therapy had a median overall survival of 37 months (95% CI: 0–79 months) and patients receiving systemic therapy only as second-line therapy had a median overall survival of 13 months (95% CI: 10–16 months; *p* < 0.001).

In a Kaplan–Meier analysis, survival differed between the three second-line treatment modalities (Figure 4, log rank *p* < 0.001). Patients receiving second-line surgery had a median overall survival of 45 (95% CI: 28–62) months, patients receiving second-line RT or systemic therapy/RT had a median overall survival of 37 (0–79) months, and patients receiving systemic therapy as second-line therapy had a median overall survival of 13 (10–16) months. Furthermore, in a Cox regression model including patient sex and age, ASA I/II vs. ASA III/IV, extent of persistence, residual vs. progressive disease, initial UICC (Union internationale contre le cancer) stage, and initial tumor site as covariates, survival differed between the three investigated second-line treatment modalities (*p* < 0.005, Table 5). Compared to surgical second-line treatment, patients receiving RT or systemic therapy/RT had a hazard of death ratio of 1.5 (0.8–3.5, *p* = 0.17) and patients receiving systemic therapy as a second-line treatment had a hazard ratio of 5.3 (2.6–10.6; *p* < 0.0001). Significant covariates in this model were ASA score (*p* = 0.015) and initial UICC stage (*p* = 0.016). Moreover, patients older than 80 years had an increased risk of death during the observation period (*n* = 4; HR 5.1, 1.1–23.0, *p* = 0.08).

## 3. Discussion

### 3.1. Study Population

We were interested in the fate of patients with incident HNC, which had persisted after first-line treatment. Patients with recurrent disease following a CR were not included in this evaluation. Persistent HNC differs from recurrent HNC in various aspects. Persistence suggests poor response behavior to radiotherapy or systemic therapy. Moreover, in persistent HNC, prior radiotherapy is frequently not long ago, prohibiting reirradiation, and surgery may be performed more easily because postradiogenic tissue fibrosis is less advanced. We analyzed the data of a carefully maintained clinical tumor registry. Data reflect real-world conditions in an oncological center in Central Europe with approximately 100 patients with incident HNC per year. First-line treatment was based on National Comprehensive Cancer Network (NCCN) guidelines [9]. Response to first-line treatment was evaluated 8–10 weeks after the end of treatment. Essentially, the same diagnostic methods were used for response evaluation as for initial diagnosis. WHO response criteria [10] were preferred over response evaluation criteria in solid tumors (RECIST) criteria [11], because they are easier to handle in everyday clinical practice. Persistent HNC was grouped into residual disease (RD), including partial response and no change, and in progressive disease (PD).

From 837 patients with incident HNC, 96 were excluded from the analysis, because no conclusive systematic treatment response evaluation was available. These exclusions were not arbitrary, but reflect real-world conditions. Excluded patients had not survived until systematic treatment response evaluation, had primarily received BSC, or had rejected any first-line treatment. Of the remaining 741 patients with systematic treatment response evaluation, 76% had CR and 24% had persistent HNC. CR and persistence were pathologically confirmed in most cases. These results are in line with recent publications, where CR rates range between 72% and 80% [12,13].

Of 175 patients with persistent disease, 90 had RD and 85 had PD following first-line treatment. Overall survival in patients with CR, RD, or PD differed substantially (Figure 2). Actuarial 5-year overall survival in patients with CR 8–10 weeks following end of first-line treatment was 73%. CR after first-line therapy is therefore considered at most a weak early surrogate marker for survival. On the other hand, persistent disease is a first-line treatment failure associated with poor survival. Particularly patients with PD following first-line treatment had a bad prognosis. Frequently, new distant metastases were detected in patients with PD (Table 2).

### 3.2. Second-Line Treatment vs. BSC

Failures of first-line treatment frequently occurred in patients with unfavorable initial disease constellation, who were nevertheless offered a chance for a possibly curative first-line therapy. A basic question to the ITB in patients with persistent HNC is if a second-line anticancer treatment is advised or if the patient should rather receive BSC. Of the 175 patients with first-line treatment failure, 63 (36%) were considered not to benefit from second-line treatment by the ITB and received BSC. These patients had unresectable persistent disease and/or were not amenable to RT. Additional factors favoring BSC instead of second-line treatment were age over 80 years, severe comorbidity, and high initial UICC stage, frequently in combination [14,15,16] (Table 3). However, individual patient or disease factors which are difficult to classify and record, such as clinical impression of treating physicians, may also have influenced ITB decisions.

At the time of treatment of these patients, PD-L1 antagonists were not yet available. Today, some patients would have been treated with PD-L1 antagonists [17] if they had been eligible for previous platinum-based therapies. However, this is often not the case. For several patients, the requirement for platinum-based therapy before PD-L1-antagonist treatment is a relevant restriction. In the recent Checkmate-141 trial, nivolumab prolonged median overall survival in patients with recurrent or metastatic HNC not amenable for surgery or RT from 5.1 months in the single agent chemotherapy comparison arm to 7.5 months [18]. Overall, this is less than under BSC treatment in the present evaluation; however, the study populations may differ in various aspects.

### 3.3. Second-Line Treatment

Second-line treatment was carried out in 112/175 patients. In patients with persistent HNS, second-line therapy had a positive effect on overall survival. Whereas almost 30% of patients with second-line treatment survived 5 years (*p* < 0.001), no patient submitted to BSC survived 5 years. Similarly, the median overall survival was worst in patients receiving BSC (10 months; 95% CI: 9 to 11 months, Figure 3). The most frequent mode of second-line treatment was surgery (53/112), in 4 patients with postoperative RT or systemic therapy/RT. Second-line surgery was limited by resectability and patient health status. CR was finally achieved in almost 80% of patients with second-line surgical treatment. The most common type of surgery was neck dissection (31/53, 57%), followed by surgery at the primary tumor site combined with neck dissection (12/53, 22%), and surgery at the primary tumor site alone (9/53, 17%). Patients with persistent cervical lymph nodes had the best benefit from surgical second-line treatment. Median survival of 31 patients with persistent disease only in the neck was 45 months. This was significantly better than in patients with persistence at any other site (*p* = 0.001). No perioperative mortality was observed. However, surgical second-line treatment of HNC at the primary site carries a high risk of morbidity. Complications like fistula formation, wound infection, or flap necrosis and impairment of speech and swallowing may occur [19]. Complication rates of second-line surgery varied between 10% and 88% and surgical mortality rates ranged between 0% and 18% [19].

RT or systemic therapy/RT was the second-line treatment in 27/112 patients and approximately half of them achieved CR (14/27). The use of second-line RT was severely restricted by recent locoregional RT in several patients. Approximately one third (8/27) of the patients in this second-line treatment group received SBRT (stereotactic body radiotherapy) for isolated pulmonary metastasis [20] or conventional RT for bone metastasis (3/27). Another frequent cause for second-line RT or systemic therapy/RT were unexpected pathological lymph nodes in a surgically treated clinical N0-neck (7/27).

Systemic therapy alone was primarily given to patients who did not qualify for surgery or RT, but qualified for systemic therapy. Combination chemotherapy was usually given according to the protocol suggested by Vermorken and colleagues and consisted of cetuximab plus platinum/5-fluorouracil [5]. Compared to platinum-based CHT plus 5-fluorouracil alone, this triple therapy was reported to improve overall survival in recurrent or metastatic squamous-cell carcinoma of the head and neck [5]. One of the 32 patients receiving second-line systemic therapy achieved CR. Of the 12 patients who had died during second-line treatment, 10 had received systemic therapy (Table 4). This is primarily a consequence of the long duration of systemic treatment.

### 3.4. Influence of Second-Line Therapy Modality on Overall Survival

Patients receiving surgery as second-line therapy had the best median overall survival (45; 28 to 62 months), followed by RT or systemic therapy/RT (37; 0 to 79 months) and systemic therapy only (13; 10 to 16 months). In a Cox regression model including sex, age at diagnosis, ASA score, tumor site, initial UICC stage, residual vs. progressive disease, and extent of tumor persistence, survival of patients with surgical second-line treatment and second-line RT or systemic therapy/RT did not differ significantly. However, patients receiving systemic therapy as a second-line treatment had a hazard ratio of 5.3 (2.6–10.6; *p* < 0.001) compared to surgical second-line treatment.

In patients with persistent HNC following first-line treatment, second-line treatment selection was governed by the treatment options, which remained after first-line treatment. This is almost the antipole of a randomized trial. Therefore, even in a multifactorial analysis, comparison of treatment results is merely descriptive. However, most authors agree that surgical salvage is the preferable second-line option for patients with resectable persistent HNC and favorable performance status [17,21,22,23]. The results of this register study are also in line with current NCCN guidelines and provide some data to support the NCCN algorithm [9]. Advances in surgical techniques often provide a better functional and esthetic outcome following second-line surgery [17,24,25]. However, extensive surgery that is often required is frequently associated with adverse events and complications, loss of function, visible deformity, high economic costs, and even death [19,23].

## 4. Materials and Methods

### 4.1. Clinical Tumor Registry

All patients with HNC treated as inpatients at the Department of Otorhinolaryngology-Head and Neck Surgery, Medical University of Innsbruck, Austria, were recorded in a clinical tumor registry. If the last follow-up was censored and older than 6 months, patients, their relatives, or attending physicians were contacted to learn about the survival status. In addition to data on initial diagnosis, treatment, and survival, the chronological sequence of the course of disease was recorded at each follow-up visit, including treatment response and second-line treatment for persistent disease. This allows a detailed analysis of the whole course over several phases of disease. All data from the tumor registry were continuously updated and checked for accuracy.

### 4.2. Outcome of First-Line Treatment

First-line treatment was recommended by an ITB in line with National Comprehensive Cancer Network (NCCN) Guidelines [9] and included surgical treatment, RT, combination or single-agent systemic therapy, including immunotherapy, and various combinations of these. All patients were routinely assessed for treatment response 8–10 weeks after the end of first-line treatment. Treatment response evaluation included contrast enhanced CT, MRI or PET-CT scans, and a restaging endoscopy, usually under general anesthesia, with biopsies from the initial tumor sites [26]. Treatment response was categorized according to WHO response criteria [10] in complete response (CR), residual disease (RD), including partial response and no change, and progressive disease (PD). Results of response evaluation were presented to the ITB. If the ITB confirmed CR, regular follow-up visits were recommended. In case of persistent HNC, second-line therapies or BSC were advised.

### 4.3. Inclusion and Exclusion

For the present evaluation, patients with HNC diagnosed between 1 January 2008 and 31 December 2016 were included. Only patients with incident HNC, except carcinomas of the thyroid gland, esophagus, eye, brain, spine, or skin, were included. Patients with distant tumors metastasizing to the head and neck region were also excluded. In a second step, patients who did not undergo conclusive systematic treatment response evaluation 8–10 weeks following end of first-line treatment were excluded. Of the remaining patients, various patient and disease parameters were recorded and categorized, including patient age and sex, American Society of Anesthesiologists Physical Status (ASA) score as a simple measure of comorbidity [27], initial tumor site and stage according to UICC classification version 7 [28], and first-line treatment. Immunohistochemistry was used to assess the p16 status [29]. It was defined positive when more than 60% of the cancer cells were stained positive; otherwise, p16 was categorized as negative [30].

### 4.4. Extent of Persistent Disease and Second-Line Treatment Protocols

Persistent disease following first-line treatment was grouped into residual disease (RD; partial response and no change) and progressive disease (PD). Site of persistence was categorized in local (at the primary tumor site), regional (regional lymph nodes), locoregional (primary tumor site and regional lymph nodes), distant (distant metastasis), and combinations of these. Treatment of persistent disease was basically divided in BSC or second-line treatment. BSC included all measures aiming to alleviate pain or other symptoms and to improve quality of life without the administration of antineoplastic therapies [31,32]. Second-line treatments were further grouped into (a) surgical treatment with or without postoperative RT or systemic therapy/RT, (b) definitive RT or systemic therapy/RT without surgery, or (c) single-agent or combination systemic therapy (CHT), including immunotherapy (IT). In line with recent NCCN guidelines, surgical treatment was favored if tumors were considered resectable [9]. Local residual tumor resection was frequently combined with free flap reconstruction. Neck dissections were either performed during resection of residual tumor or as a sole treatment if persistent disease was limited to the neck. Postoperative RT or systemic therapy/RT was given if a sufficient radiation dose could be applied and was recommended by the ITB. RT modalities complied with NCCN guidelines [9]. Systemic therapy/RT was either performed as single-agent CHT with Cisplatin 25 mg/m^2^ weekly [33] or Cetuximab at a loading dose of 400 mg/m^2^ a week before RT, followed by 250 mg/m^2^ weekly during and after RT [34] or as a combination systemic therapy with mitomycin C/ 5-fluorouracil 10 mg/m^2^/1000 mg/m^2^ [35]. CHT alone was generally performed according to the combination therapy protocol suggested by Vermorken and colleagues (5-FU, cis-/carboplatin, Cetuximab) [5]. Alternatives were cis-/carboplatin/5-FU, docetaxel, or Cetuximab as single-agent therapy [36].

### 4.5. Data Analysis

A positive vote from the Ethics Committee of the Medical University of Innsbruck was obtained (AN2017-007937 1/4.31) on 7 June 2017. An informed consent from the patients was not considered necessary by the Ethics Committee as it is a retrospective study. Median follow-up time was calculated as proposed by Schemper et al. [37]. Frequencies were tabulated and compared with Chi-square tests. For metric data, mean and standard error are provided unless stated otherwise. Overall survival was analyzed with the Kaplan–Meier method and compared with log rank tests. Actuarial (life table) analysis was used to calculate 5-year survival. In addition, overall survival was analyzed with Cox regression models. Backward elimination using a likelihood ratio with a *p*-value of 0.05 for inclusion and 0.1 for exclusion was used. If a group could serve as a plausible control, indicator coding was used; otherwise, deviation coding. Statistical analyses were performed using SPSS 24 (IBM Corporation, Armonk, NY, USA).

## 5. Conclusions

In unselected HNC patients, first-line treatment failure was observed in approximately 25%. In approximately 1/3 of patients with persistent HNC, best supportive care was advised. Second-line treatment resulted in CR in approximately 50% and significantly improved survival. In line with NCCN guidelines for persistent HNC, surgical second-line therapy resulted in best survival. Stereotactic body radiotherapy was highly effective in treating HNC progression at distant sites. We think that early treatment response should be performed in every HNC patient. Treatment response evaluation allows early initiation of second-line treatment, which offers patients with persistent disease a realistic chance of achieving complete response and long-term survival after all.

## Figures and Tables

**Table 1:** Characteristics of 175 patients with persistent HNC after first-line treatment. All patients with incident HNC treated between 2008 and 2016 were consecutively included. To assess first-line treatment response, patients received a thorough diagnostic re-evaluation 8–10 weeks after completion. Abbreviations: ASA: America society of anesthesiologists, IHC: immunohistochemistry.

Variable	Value	Count	Percent
Sex	Male	133	76%
	Female	42	24%
Age at diagnosis	≤50	25	14%
	51–60	51	29%
	61–70	52	30%
	71–80	30	17%
	>80	17	10%
ASA I/II vs. ASA III/IV	ASA I/II	40	37%
	ASA III/IV	68	63%
Common tumor sites	Lip/oral cavity	32	18%
	Oropharynx	53	30%
	Hypopharynx	23	13%
	Larynx	34	19%
	Other	33	19%
Clinical stage	Stage 1	9	5%
	Stage 2	12	7%
	Stage 3	18	10%
	Stage 4a	105	60%
	Stage 4b	18	10%
	Stage 4c	13	7%
p16-IHC	Negative	90	80%
	Positive	22	20%
First-line treatment	Surgery only	23	13%
	Surgery and postoperative radiotherapy	15	9%
	Surgery and systemic therapy/radiotherapy	20	11%
	Systemic therapy/radiotherapy	73	42%
	Chemotherapy	11	6%
	Radiotherapy	26	15%
	Radioimmunotherapy	7	4%
First-line treatment adherence	Treated as planned	140	80%
	Discontinued	19	11%
	Treatment modified	16	9%

**Table 2:** Site and extent of persistent HNC after first-line therapy. Residual disease included partial response and no change according to WHO response criteria. Progressive disease frequently occurred at distant sites.

RD/PD	Site of Persistence					Total
	Primary Site	Primary Site and Neck	Neck Only	Distant Only	Distant and Primary Site and/or Neck	
Residual disease	44	10	28	3	5	90
Progression	30	5	4	20	26	85
Total	74	15	32	23	31	175

**Table 3:** Patients and disease factors (left column) and frequencies of BSC vs. any second-line antineoplastic treatment in patients with persistent HNC after first-line therapy. In brackets, row percent for each factor value are presented. Only factors with unequal frequency distribution (Chi-square *p* < 0.05; right column) are tabulated. Sex, tumor site, and initial clinical union internationale contre le cancer (UICC) stage did not significantly influence whether patients received BSC or second-line treatment. All treatments were based on the advice of the interdisciplinary tumor board.

Variable	Value	Second-line	BSC	Total	*p*-Value
Age at diagnosis	≤50	20 (80%)	5 (20%)	25	0.001
	51–60	38 (75%)	13 (25%)	51	
	61–70	33 (63%)	19 (37%)	52	
	71–80	17 (57%)	13 (43%)	30	
	>80	4 (24%)	13 (76%)	17	
ASA I/II vs. ASA III/IV	ASA I/II	34 (85%)	6 (15%)	40	0.004
	ASA III/IV	40 (59%)	28 (41%)	68	
p16-IHC	Negative	58 (64%)	32 (36%)	90	0.047
	Positive	19 (86%)	3 (14%)	22	
First-line treatment	Surgery only	19 (83%)	4 (17%)	23	<0.001
	Surgery and PORT	8 (53%)	7 (47%)	15	
	Surgery and systemic therapy/RT	13 (65%)	7 (35%)	20	
	Systemic therapy/RT	56 (77%)	17 (23%)	73	
	Systemic therapy	5 (45%)	6 (55%)	11	
	Radiotherapy	8 (31%)	18 (69%)	26	
	Radioimmunotherapy	3 (43%)	4 (57%)	7	
First-line treatment discontinuation	No	100 (71%)	40 (29%)	140	<0.001
	Discontinued	4 (21%)	15 (79%)	19	
	Treatment modified	8 (50%)	8 (50%)	16	
RD/PD	Residual disease	72 (80%)	18 (20%)	90	<0.001
	Progression	40 (47%)	45 (53%)	85	
Sites of persistence	Primary site	39 (53%)	35 (47%)	74	0.002
	Primary site and neck	12 (80%)	3 (20%)	15	
	Neck only	28 (88%)	4 (13%)	32	
	Distant only	17 (74%)	6 (26%)	23	
	Distant and primary site and/or neck	16 (52%)	15 (48%)	31	

ASA: American Society of Anesthesiology; BSC: Best supportive care; IHC: Immunohistochemistry.

**Table 4:** Complete response (CR) rates to second-line treatment in 112 patients with persistent HNC following first-line treatment depending on second-line treatment modality. (RT: Radiotherapy, RCHT: Radiochemotherapy, RIT: Radioimmunotherpay).

	Surgical Treatment ^(2)^	RT/RCHT/RIT Without Surgery	Chemotherapy Only	Total ^(3)^
With CR	41	14	1	56
No CR	12	11	20	43
Death before end of treatment ^(1)^	0	2	10	12
Total	53	27	32	112

(1) Due to tumor disease; (2) With postoperative RT or RCHT in 4 patients; (3) One patient lost to follow-up.

**Table 5:** Multivariate logistic regression model: Influential patient factors for overall survival after second-line treatment. (RT: Radiotherapy, RCHT: Radiochemotherapy, CHT: Chemotherapy, ASA Score: American Society of Anesthesiologists).

Patient Factors		Regression Coefficient B	Wald	df	Sig.	Adjusted OR	95% Confident Interval for OR	
							Lower	Upper
extent of persistence	extent of persistence		1.26	4	0.87			
	local	0.38	0.35	1	0.55	1.46	0.42	5.13
	locoregional	−0.16	0.06	1	0.81	0.86	0.25	2.96
	regional	−0.02	0.00	1	0.99	0.99	0.23	4.27
	distant	0.153	0.06	1	0.80	1.17	0.36	3.82
	locoregional and distant *	0.00				1.00		
residual vs. progressive disease	residual disease	0.16	0.15	1	0.7	1.18	0.51	2.68
	progressive disease *	0.00				1.00		
UICC stage	UICC Stage		2.61	3	0.46			
	I	−1.01	0.85	1	0.36	0.37	0.043	3.12
	II	−0.91	1.12	1	0.29	0.40	0.08	2.17
	III	−1.08	1.98	1	0.16	0.34	0.08	1.53
	IV *	0.00				1.00		
initial tumor site	tumor site		2.66	4	0.62			
	lips/oral cavity	−0.19	0.06	1	0.82	0.82	0.16	4.33
	oropharynx	−0.53	0.57	1	0.45	0.59	0.15	2.33
	hypopharynx	0.10	0.02	1	0.90	1.10	0.23	5.26
	larynx	−0.22	0.07	1	0.79	1.24	0.25	6.2
	Other *	0.00				1.00		
second-line treatment modality			9.57	2	0.01			
	surgery	−1.75	9.43	1	0.002	0.17	0.06	0.53
	RT/RCHT	−1.13	3.24	1	0.07	0.32	0.09	1.11
	CHT *	0.00				1.00		
age	age		5.23	4	0.26			
	≤50	−2.27	2.82	1	0.09	0.10	0.01	1.47
	51–60	−2.78	4.55	1	0.03	0.06	0.05	0.80
	61–70	−2.38	3.26	1	0.07	0.09	0.01	1.23
	71–80	−2.54	3.31	1	0.07	0.08	0.01	1.22
	>80 *	0.00				1.00		
ASA score	I/II	−0.9	5.88	1	0.02	0.4	0.20	0.84
	III/IV *	0.00				1.00		

OR = Odds Ratio; * category taken as reference group.
